# Normalizing the brain connectome for communication through synchronization

**DOI:** 10.1162/netn_a_00231

**Published:** 2022-07-01

**Authors:** Spase Petkoski, Viktor K. Jirsa

**Affiliations:** Aix-Marseille University, Inserm, INS, Institut de Neurosciences des Systèmes, Marseille, France

**Keywords:** Synchronization, Time delays, Normalized connectome, Spectral activation patterns, Oscillations, Resting-state networks

## Abstract

Networks in neuroscience determine how brain function unfolds, and their perturbations lead to psychiatric disorders and brain disease. Brain networks are characterized by their connectomes, which comprise the totality of all connections, and are commonly described by graph theory. This approach is deeply rooted in a particle view of information processing, based on the quantification of informational bits such as firing rates. Oscillations and brain rhythms demand, however, a wave perspective of information processing based on synchronization. We extend traditional graph theory to a dual, particle-wave, perspective, integrate time delays due to finite transmission speeds, and derive a normalization of the connectome. When applied to the database of the Human Connectome Project, it explains the emergence of frequency-specific network cores including the visual and default mode networks. These findings are robust across human subjects (*N* = 100) and are a fundamental network property within the wave picture. The normalized connectome comprises the particle view in the limit of infinite transmission speeds and opens the applicability of graph theory to a wide range of novel network phenomena, including physiological and pathological brain rhythms. These two perspectives are orthogonal, but not incommensurable, when understood within the novel, here-proposed, generalized framework of structural connectivity.

## Author Summary

All networks are composed of nodes and links, forming the structural frame, in which communication occurs. We demonstrate that graph theoretical tools make the implicit assumption of information transmission via exchange of bits, suggesting that the stronger connected nodes have greater impact upon the remainder of the network. This corollary does not extend to communication via oscillations, which is the prominent information carrier in brain networks. We extend traditional network analysis to the oscillatory domain and derive a novel network normalization complete with descriptive metrics. Along the prototypical example of the brain as a network, we illustrate the consequences of this novel approach and demonstrate that the normalization robustly explains the emergence of the prominent frequency-specific network cores, which cannot be understood within the traditional framework.

## INTRODUCTION

Network theory significantly advanced our understanding of complex processes in nature, ranging from gene regulation of protein interactions (Maslov & Sneppen, 2002) and coordination of brain activity (Bassett & Sporns, 2017) to social networks (Mislove, Marcon, Gummadi, Druschel, & Bhattacharjee, 2007). Connectivity is the dominant concept that shapes the capacity of a network to transmit information (E. Bullmore & Sporns, 2012) and is described by its topological and statistical properties (Albert & Barabasi, 2002). In neuroscience, network theory has been applied on several scales, including microscopic neocortical microcircuitry (Markram et al., 2015), macroscopic structural connectivity connectome (Sporns, Tononi, & Kötter, 2005), and networks of coordinated brain activity, also called functional connectivity (Friston, 2011). Models of brain networks based on connectomes (Sanz-Leon, Knock, Spiegler, & Jirsa, 2015) have demonstrated individual predictive power in resting-state paradigms (Melozzi et al., 2019), cognitive tasks (Brovelli et al., 2004), and brain disease such as epilepsy (Proix, Bartolomei, Guye, & Jirsa, 2017). In all these applications, there remains a deeply rooted understanding of signal transmission in which bits of information are transmitted between nodes, independently of the underlying oscillations and brain rhythms. But just as the particle picture is complemented by a wave picture in physics, similarly in neuroscience there are oscillatory processes deeply implicated in healthy and pathological brain activation (Buzsáki, 2009), such as cognitive functions (Haegens, Nácher, Luna, Romo, & Jensen, 2011) or aberrant discharges in epilepsy (Proix et al., 2017). How such rhythms support the communication through phenomena such as phase and frequency dependance, does not have a fundamental description within the particle view of information processing, which describes network processes by activations, but does so in the wave picture that imposes a language in terms of synchronization.

The importance of the particle-wave duality in network neuroscience becomes evident when considering signal transmission delays, present in large-scale brain networks and ranging on the order of 10 to 200 ms (Nunez & Srinivasan, 2006). As physiological rhythms in the brain lie in the same range (Buzsáki & Draguhn, 2004), shifts in arrival times due to delays have minimal to no effects in the particle view, but result in frequency-dependent effects in the wave view, where a synchronized pair of oscillators may switch from full synchrony to anti-synchrony. As such, frequency and time delays become inseparable properties of the network and together with the connectome determine the synchronization and nodal activity (Petkoski & Jirsa, 2019; Petkoski, Palva, & Jirsa, 2018). This also confirms previous computational studies about the effects of the space-time structure of the brain on its emergent dynamics (Ghosh, Rho, McIntosh, Kötter, & Jirsa, 2008), and could also determine the spectrally dependent information processing capacities based on synchrony (Fries, 2015).

Current state-of-the-art network neuroscience is mainly focused on the topological aspects in describing the connectivity of the brain (Bassett & Sporns, 2017; Bassett, Zurn, & Gold, 2018). By omitting space and physical distances between interacting units, it limits itself to the particle view (Rubinov & Sporns, 2010). Possible exceptions from this are the cases in which structural network motifs are shown to be related to the functional hubs due to the time delays (Gollo, Zalesky, Matthew Hutchison, van den Heuvel, & Breakspear, 2015), but without spectral specificity. In rare cases, when lengths of the links are studied (Roberts et al., 2016), it is still done in static manner, without consideration for the impact of the delays on the emerging dynamics, thus critically demanding the extension of network neuroscience to the wave picture. The only attempts to study negative links between brain regions are limited to the clustering of functional data with negative correlations (Rubinov & Sporns, 2010), without any application to the structural links, for which there has never been any mechanistic explanation that would effectively render them inhibitory for the synchronization.

## RESULTS

### Particle Versus Wave Representation for Network Dynamics

**Figure 1.**
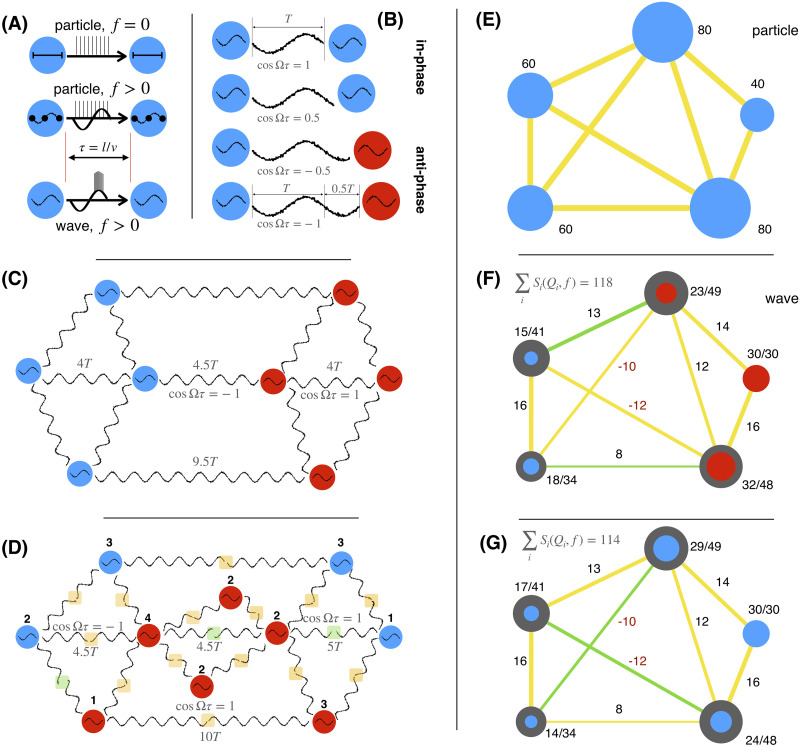
Spatiotemporal network organization and wave coupling weights for synchronized networks. (A) Particle (static or frequency-independent) and wave (synchronization-dependent) interactions with delay *τ*, due to distance *l*, and propagation velocity *v*. Top: Transmission of packets over non-oscillatory local dynamics. Middle: A particle type of transmission that does not depend on the coherence (e.g., during strong perturbation) and is thus independent of the underlying oscillatory dynamics. Bottom: The communication is dependent on the synchronization. (B–D) In- and anti-phase synchronization for fixed frequency and different time delays for two oscillators (B), and for networks with a resonant (C) and non-resonant (D) spatiotemporal alignment of the oscillators. Nodes with same color are in-phase with each other, and anti-phase with others, as also illustrated with the waveform. Time delays are shown relative to the period of oscillations *T*, and the normalization factor is shown for some of the links. (D) Midpoints of links with in-phase oscillations are highlighted in yellow, and in green if anti-phase, and the sum of all positively and negatively contributing links (yellow minus green links) to each node is also shown. (E–G) Wave coupling weights for a particle (or static) case (E) and for optimal (F) and suboptimal (G) phase arrangements of nodes. Links contributing positively to the synchronization are yellow, and negative contributions are green. Lines have width proportional to $|w_{ij}^{\left(f\right)}|$ (shown next to the links), and the size of the circles corresponds to the nodes’ spectral capacity (dark) and strength (colored), both shown for each node.

The particle-wave dichotomy for network communication appears in network dynamics because of the finite transmission velocities for the signals between spatially distributed nodes. The governing equation reads

$${\dot{x}}_{i}=F\left(x_{i}\right)+\sum_{j}w_{ij}h\left(x_{j}\left(t-\tau_{ij}\right)\right),$$ (1)

where ***x***_*i*_ is a vector of states, *F* is a nonlinear function, and *h* is the coupling function. Within the particle view, ***x*** is a one-dimensional scalar variable such as firing rate, rendering the interactions to be frequency-independent; see Figure 1A. Consequently, the strongest flow of information and the activity is generally along the links with the largest weight (E. T. Bullmore & Sporns, 2009), although there are cases in which hubs are significantly more influential (Novelli, Atay, Jost, & Lizier, 2020; Timme et al., 2016). In addition, time delays are less important during single events that occur independent of the possible underlying oscillatory activity (middle plot of Figure 1A), such as the action potentials that make up subsequent metrics such as firing rate. Similar examples of particle-like interactions happen during strong perturbations and stimulations (Spiegler, Hansen, Bernard, McIntosh, & Jirsa, 2016), or when the coupling is no longer weak and bifurcations occur, such as during seizure propagation in epilepsy (Olmi, Petkoski, Guye, Bartolomei, & Jirsa, 2019).

Networks of oscillators, on the other hand, are often used to study dynamical systems for which the local activity is multidimensional and nonlinear (Pikovsky, Rosenblum, & Kurths, 2001). They have been conceptualized to be responsible for the communication in the brain through coherence (Fries, 2015) and synchronization (Palva & Palva, 2012; Figure 1A). However, this aspect is still unlinked on the network level to weights and time-delays of the structural that shape the synchronization of the brain network (Petkoski & Jirsa, 2019). For weak couplings, dynamics of the nonlinear oscillatory system in Equation 1 are captured by phase models, of which the simplest and the most elaborate is the Kuramoto model (Kuramoto, 1984). For synchronization at frequency Ω, the model reads (Izhikevich, 1998)

$${\dot{\theta }}_{i}=\omega_{i}-\sum_{j}w_{ij}\sin\left(\theta_{i}-\theta_{j}+\Omega \tau_{ij}\right),$$ (2)

where *θ*_*i*_ denotes the phase of the *i*th node oscillating with a natural frequency *ω*_*i*_. Note that if time delays were short so that they would not appear explicitly in the phase model (Izhikevich, 1998), then Equation 2 would have still represented the phase model for the case of synchronization.

From Equation 2, when delays are negligible compared with the timescale of the system, the interactions are governed only by the weights, as in the particle case. Because of better tractability and the readily applicable tools from graph theory, this has been the default network representation in neuroscience. However, when the time delays are comparable to the timescale of the intrinsic oscillations, they need to be included in the analysis of network dynamics. This system can only be solved numerically, in order to unveil the activation patterns at different frequencies, as there is no alternative graph theoretical approach that integrates the topology and the impact of the time delays on the oscillations. Hence the systematic effect of the time delays on the emergent dynamics in Equation 1 is concealed, beyond the synchronizability and the order parameter that can be calculated using the Ott and Antonsen ansatz (Ott & Antonsen, 2008), but only for all-to-all couplings and spatially decomposed delays (Petkoski et al., 2016).

To go beyond the static representation of networks, we use the insight that the impact of the direct link in the phase difference between oscillators can be separated from the rest of the network (see the Model and Methods section), leading to solutions given as

$${\Delta \theta }_{ij}=\sin^{-1}\frac{\Delta \omega_{ij}-I_{ij}}{w_{ij}\cos\Omega \tau_{ij}}.$$ (3)

Here Δ*ω*_*ij*_ = (*ω*_*i*_ − *ω*_*j*_)/2, and normalized wave coupling $w_{ij}^{\left(f\right)}$ = *w*_*ij*_ cosΩ*τ*_*ij*_ describes the impact of the direct link between the oscillators. The influence of all the other links of these two nodes is contained in *I*_*ij*_. The cosine term vanishes for particle-like communication Ω*τ*_*ij*_ → 0, simplifying Equation 3 to the case of no. For interactions of synchronized oscillations, wave coupling $w_{ij}^{\left(f\right)}$ unifies spatiotemporal aspects of each link by modulating weights with the frequency-specific impact of the time delays. This reflects duality in the interactions, which in the wave case is dependent on the timings of the waveforms arriving from the distant nodes; see Figure 1B–D.

As illustrated in Figure 1B, two delay-coupled oscillators can synchronize either in-phase or anti-phase (Petkoski et al., 2018; Pikovsky et al., 2001), depending on the sign of cosΩ*τ*. The former is stable for positive wave couplings, the latter for negative. The same is true for networks (Petkoski et al., 2018) and not limited to phase oscillators (Petkoski & Jirsa, 2019). Time delays can be perfectly spatially distributed to cause maximum synchronization at a given frequency (Figure 1C), but a more realistic scenario is when their distribution causes some links to decrease the synchronization, that is, if $w_{ij}^{\left(f\right)}$ < 0 for in-phase nodes, or if $w_{ij}^{\left(f\right)}$ > 0 for anti-phase. One possibility here is the oscillators rearranging their phases to minimize this disturbance (Figure 1D). From here, to identify the skeleton of the wave synergy we define a spectral strength and capacity for each node

$$S_{i}\left(f,Q_{i}\right)=\sum_{j\in Q_{i}}w_{ij}^{\left(f\right)}-\sum_{j\notin Q_{i}}w_{ij}^{\left(f\right)},$$ (4)

$$C_{i}^{\left(f\right)}=\sum_{j}|w_{ij}^{\left(f\right)}|.$$ (5)

In the static case, both metrics yield the topological node strength, Figure 1E. *S*_*i*_(*f*, *Q*_*i*_) is adapted from the particle case node strength (Rubinov & Sporns, 2010) by accounting for possible inhibitory contribution of some links to the overall synchronization, that is, negative wave coupling from an in-phase node, or a positive one from an anti-phase node; see Figure 1D, F, and G. The cluster *Q*_*i*_ thus contains all the nodes that are in-phase with the node *i*. Related to this, $C_{i}^{\left(f\right)}$ gives the upper bound of node’s synchronizability and hence of its *S*_*i*_(*f*, *Q*_*i*_); see Figure 1F and G. Therefore, it shows the strength of the node when all its links contribute positively to its dynamics. As shown in the same plots, different phase arrangements of the nodes change their spectral strength. A hub node can become more peripheral, or vice versa, while still being constrained by its spectral capacity, which does not change as different arrangements are realized for a fixed frequency.

For simplicity, in the rest of the manuscript we will omit the explicit dependence of the spectral strength on the node-specific cluster *Q*_*i*_, but instead we will refer to specific in-/anti-phase arrangements on the network level that imply specific clustering for the nodes. For example, in most of the results we will show results for $S_{i}^{\left(f\right)}$ from a clustering that maximizes its sum over the whole network.

### Normalized Connectome and Spectral-Dependent Activity of Brain Regions

**Figure 2.**
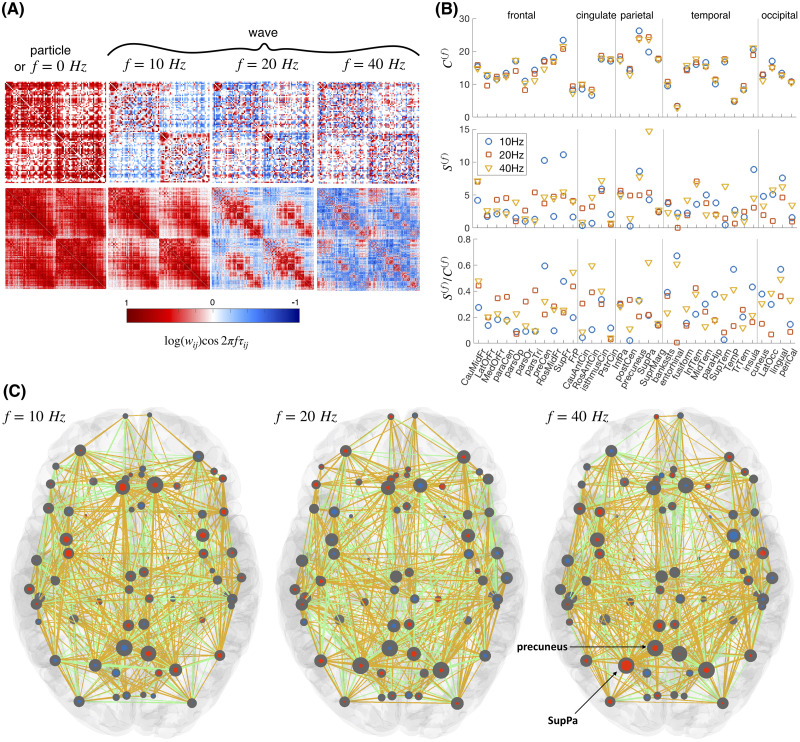
Normalized connectomes at different frequencies. (A) Wave couplings for a human (top) and averaged mice (bottom) connectomes. (B) Spectral strength and capacity, and their ratio for regions of the left hemisphere ordered by lobes for three different frequencies. Full names of the regions are shown in Table S14 in the Supporting Information. (C) Spectral strength (blue or red, indicating anti-phase arrangements) and capacity (gray) for cortical regions of a human brain. The links with positive contribution to the optimal phase arrangement are yellow, and others are green (cf. Figure 1B). At each frequency only the links with spectral strengths above 0.33 of the mean strength are shown for simplicity.

The wave coupling weights are frequency-dependent, and in the case of the connectome this causes activations of different cores. We assume homogeneous propagation velocity, which is often used as a first approximation, resulting in time delays being defined by the lengths of the links (Ghosh et al., 2008; Petkoski & Jirsa, 2019; Petkoski et al., 2018; Sanz-Leon et al., 2015), that is, *τ*_*ij*_ = *l*_*ij*_/*v*. The cosine term modulates the strength of the links, which can even change the sign, reverting once positive interactions depending on the frequency. This is demonstrated in Figure 2A for human (Van Essen et al., 2013) and mice (Oh et al., 2014) connectomes.

**Figure 3.**
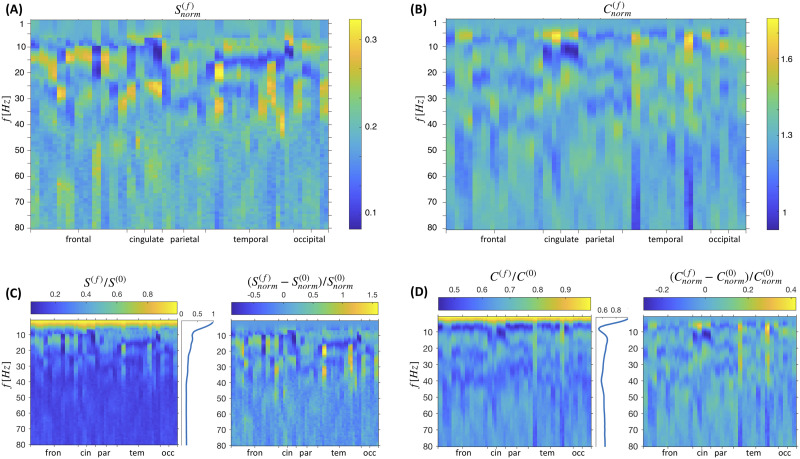
Spectral strength and capacity for brain regions. Mean values are shown over 100 subjects and for frequencies in a step of 1 Hz. Brain regions are ordered by lobe, with exact ordering within lobes given in Table S14 in the Supporting Information, and each right hemisphere region is placed right after its left hemisphere counterpart. (A, B) *S*^(*f*)^ and *C*^(*f*)^ normalized across each frequency, and (C, D) their ratio and difference compared with the case with no delays, where the former is for the non-normalized metrics, to reveal the frequency-dependent mean activity.

The spectral strength and capacity are illustrated in more detail in Figure 2B and C for three different frequencies, and they are shown for all the brain regions ordered by lobes in Figure 3. The phase arrangement of the nodes is obtained by maximizing the overall spectral strength of the network (see the Model and Methods section for more details). For example, the two metrics in Figure 2B and C illustrate that the highest spectral capacity for regions of cingulate are at 20 Hz and their strength is lowest at 10 Hz, while the occipital lobe has highest spectral strength and capacity at 10 Hz. Large spectral capacity implies possibility for a given node to get greater strength during different phase arrangements. As illustrated in Figure 1B, even for a network of several nodes it can already become impossible not to have negatively contributing links that inhibit the synchronization. The ratio $S_{i}^{\left(f\right)}$/$C_{i}^{\left(f\right)}$ shows the proportion of the weighted links that contribute positively to the spectral strength of the node in the optimal phase arrangement. Hence, the superior parietal (SupPa) region is by far the strongest at 40 Hz because of the more positively contributing links, as compared with the precuneus, which is much weaker at this arrangement, besides having a similar capacity.

In Figure 3, relative activation patterns of all the brain regions are shown for the frequencies up to 80 Hz. They are more heterogeneous for the frequencies between alpha and lower gamma, and homotopic brain regions generally show similar activation patterns, because of the high symmetry of the weights and delays in the connectome. Frequency-normalized values of the spectral strength and capacity unveil the regions with strongest relative activity at each frequency, whereas the relative change of the activation compared with the particle case is also shown (right plots in Figure 3C and D). The strength of each region’s activity relative to the particle case as well as the mean relative activity for the whole brain are shown in the left plots in Figure 3C and D. For every region the strength generally decreases with frequencies, as it is clearly seen in their mean, while the capacity has much steeper decline up to alpha frequencies, when it slightly recovers and stays largely constant.

### Spectral-Dependent Activity of RSNs and Brain Lobes

**Figure 4.**
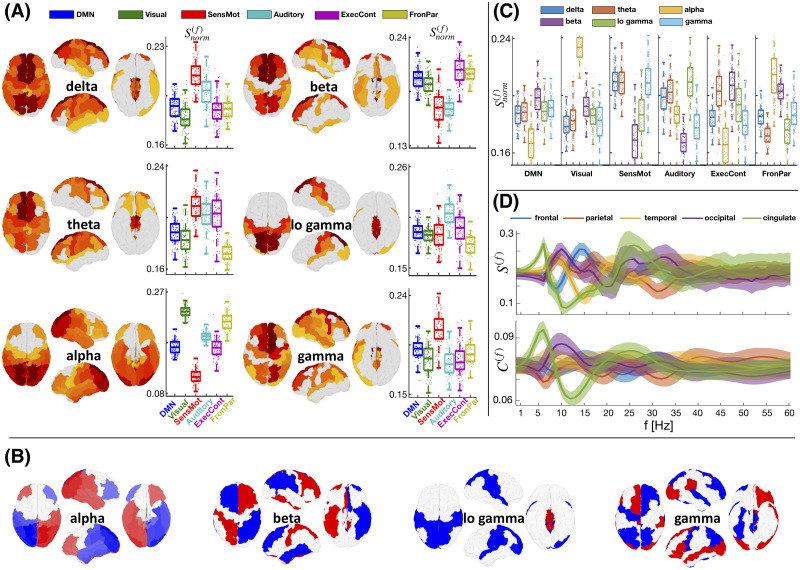
Cortical activation patterns of 100 subjects for different frequencies. (A) Mean spectral strength is shown for different cortical regions (higher for warmer colors) and frequency bands (see Table S13 in the Supporting Information), and activation of resting-state networks (RSNs) is projected in the frequencies. (B) In-/anti-phase arrangements of mean $S_{i}^{\left(f\right)}$ are indicated with red and blue. (C) Relative contribution of the frequency bands per RSN. Boxplots contain the scatterplots of all 100 subjects. (D) Mean spectral strength and capacity (lines) and regions of two standard deviations (shaded) calculated in step of 1 Hz for each lobe. The values per given RSN and lobe are normalized by the number of regions in that subnetwork. Brain regions of different RSNs are given in Table S15, and EEG frequency bands are given in Table S13 in the Supporting Information.

Studies of functional connectivity have demonstrated that signals in brain regions that are related in different tasks can also correlate even in the absence of an apparent task, hence the notion of resting-state networks (RSNs). The most consistent RSNs are default mode network (DMN), visual, sensory/motor (SensMot), auditory, executive control (ExecCont), and frontoparietal (FronPar; Damoiseaux et al., 2006). In Figure 4, we show the average spectral strength and capacity of brain regions of 100 subjects, further projected on RSNs and anatomical lobes. Regions with the strongest activity averaged over the subjects are shown in Figure 4A, through the mean $S_{i}^{\left(f\right)}$, which is normalized at different frequency bands. The same panel contains the boxplots of the strengths of the RSNs of different subjects. Panel B of Figure 4 contains the same metric, but the in-/anti-phase arrangements of the nodes are taken into account through the sign of $S_{i}^{\left(f\right)}$, which is now plotted in red-white-blue color code, with red regions being anti-phase with the blue. In Figure 4C, the strength at each RSN is projected across the bands, complementing the information about the spectral affinity of the RSN. For example, the visual network is by far the most active during alpha frequencies (Figure 4C), even though the alpha frequency also contains a strong activity of the frontoparietal network (Figure 4A). Figure 4D shows the mean and the standard deviation over the 100 subjects of the spectral strength and capacity of each lobe normalized at each frequency. It shows that the robustness over the subjects is much stronger for lower frequency bands (up to around 30 Hz), similar to the most robust empirically observed spectral activation patterns (de Pasquale, Corbetta, Betti, & Della Penna, 2018; de Pasquale et al., 2010; Hillebrand et al., 2016; Vidaurre et al., 2018).

Some of the most remarkable and statistically significant (see Tables S1–S12 in the Supporting Information) activation patterns predicted by the metrics in Figure 4 are in agreement with the strongest empirically observed phenomena. It is due to the frequency-dependent, normalized links, that visual areas, mainly located in the occipital lobe, become the most active at alpha frequencies, as it is experimentally observed during rest with closed eyes (Mantini, Perrucci, Del Gratta, Romani, & Corbetta, 2007; Samogin et al., 2020). Similarly, while activity is dominant in the delta/theta frequencies for the anterior-cognitive state, the posterior-cognitive state is dominated by alpha frequencies (Vidaurre et al., 2018). By contrast, the sensory motor and auditory networks are more active at gamma (Cheyne & Ferrari, 2013), while DMN, which is mainly in the frontal lobe, is more prominent at the beta band (Laufs et al., 2003), associated with the idleness of the cognitive and motor setup.

### Numerical Confirmation for Forward Modeling and Linear Stability Analysis with Stuart-Landau Oscillators

**Figure 5.**
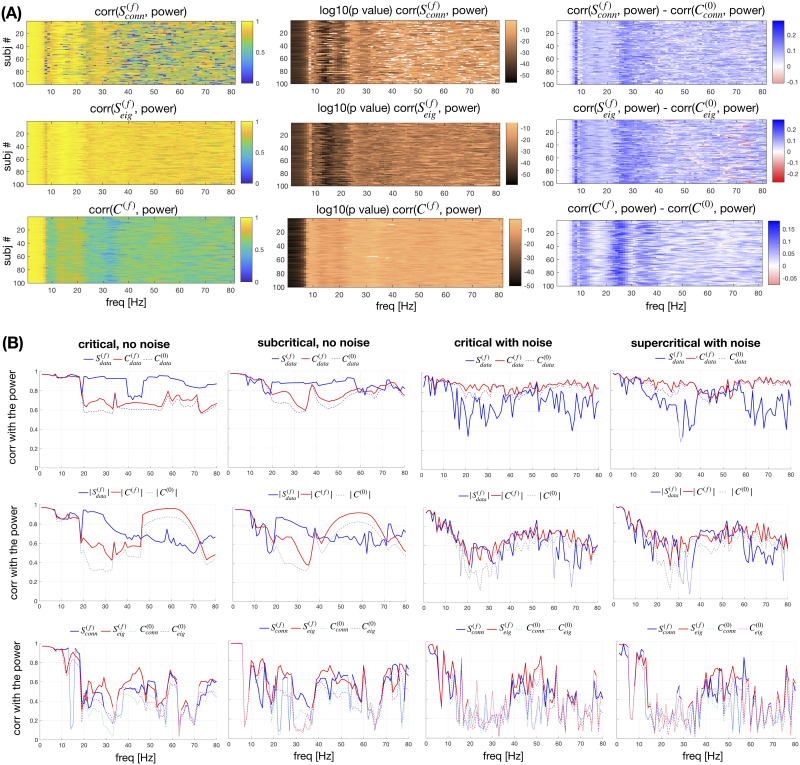
Comparison with forward models based on the normalized and the full connectome. The power across regions from the forward model is compared with the spectral capacity and strength, where the latter is calculated either using the signs of the eigenvectors or using arrangements that maximize its value, and with the null metrics assuming particle-like interactions. The power is calculated (A) using linear stability analysis (LSA) of the brain network model at criticality, constructed over the normalized connectome or (B) with numerical simulations performed for the full connectome, with the brain network model being subcritical (*G* = 17, *D* = 0) and supercritical (*G* = 9, *D* = 1e−5), and at the criticality with noise (*G* = 9, *D* = 1e−5) and without (*G* = 9, *D* = 0). (B) For the full simulations, the most prominent phase arrangement of the simulated data is also used to set the in-/anti-phase arrangements. Correlations of the powers across frequencies (A) for all 100 subjects are shown, as well as the *p* values, and the difference with the correlations assuming particle-like interactions, and (B) for one subject. Correlations with *p* < 0.01 are transparent in the first two columns of (A), and thinner lines for (B).

The activation patterns predicted by the metrics of the normalized connectome are numerically validated with a linear stability analysis (LSA) and a forward simulation of brain network model of Stuart-Landau oscillators (Petkoski & Jirsa, 2019; Pikovsky et al., 2001) over the same 100 human connectomes (Figure 5). The model is explored at critical, subcritical, and supercritical regimes, along with noise (see the Model and Methods section). For consistency, in the simulated results, powers get signs that are based on the in-/anti-phase arrangements of the simulated data, which are obtained from calculating the average phase difference between the regions and checking whether it is larger than *π*. Similarly, for consistent comparison between the metrics, the signs from the phase arrangements either from the data, from the connectome, or from the eigenvalues are explicitly assigned to the spectral strengths and capacities. Thus, for instance, the spectral strengths respectively become $S_{\text{data}}^{\left(f\right)}$, $S_{\text{conn}}^{\left(f\right)}$, or $S_{eig}^{\left(f\right)}$.

With the generative model we confirm that these spectral patterns of activity are predicted by the two graph theoretical metrics on the normalized connectome, that is, the spectral strength and capacity. The former depends on the actual phase arrangement of the oscillators. For this, besides the arrangement that maximizes the overall *S*^(*f*)^ (that is based only on the normalized connectome), we also use the signs of the eigenvectors to inform the spectral strength metric about the in-/anti-phase organization for the specific model. In this dynamical setting the amplitude and the sign of the observed activation pattern for the network coupled through the normalized connectome are guaranteed to be fully captured by the values of the first eigenvector of the LSA (Ghosh et al., 2008; Olmi et al., 2019). The signs of the different directions of the eigenvector capture the in-/anti-phase arrangements of the nodes, with the opposite-sign regions corresponding to anti-phase synchronized regions.

In Figure 5A, we show the correlation of the leading eigenvectors with spectral strength, calculated using both arrangements, for all 100 subjects. The arrangement implied by the signs of the eigenvectors takes into account the specificity of the model, and hence this case better predicts the activation of a given node. This means that the network is not always arranged in such a manner to yield maximal possible synchronization. The patterns are similar across subjects, and for frequencies larger than 40 Hz, there seem to be specific frequencies for different subjects, for which the arrangement of the signs of the eigenvectors is quite different from those maximizing *S*^(*f*)^. Spectral capacity on the other hand, has generally lower predictability than $S_{\text{conn}}^{\left(f\right)}$, but it is never insignificant or lower than the case without normalization. As shown in the third column of panel A, all three spectral metrics exhibit much better correlation than the null metric, which is constructed assuming particle-like interactions at 0 Hz. Thus, only the weights of the links are considered, giving the in-strength of the nodes $C_{i}^{\left(0\right)}$ = ∑_*j*_ *w*_*ij*_. Since the null model cannot yield anti-phase nodes, for more conservative comparison we assign it signs predicted by the arrangements of the nodes with which it is being compared.

In Figure 5B, the predictivity of the metrics is tested by comparing them with the power obtained by simulating the model built with the same oscillators (Equation 17), but over the full connectome containing both weights and delays (Equation 18). Moreover, this is extended for the subcritical and supercritical regimes of the system, along with noise. The comparison shows that each of the wave interactions metrics has consistently higher predictability than its particle-like counterpart across all the frequencies. This holds even for noisy systems far from the criticality, where the correlation is generally larger than 0.5 if the phase arrangements are taken from the data. It is worth noting that for the full system the spectral capacity seems to be more informative than the spectral strength, because the dynamics often become nonstationary and multi-stable because of the explicit time delays (Petkoski et al., 2016). Nodes might switch from in-phase to anti-phase and back, and different phase arrangements lead to different spectral strengths over time. As a result, each node’s average relative strength within the network might be better captured by its capacity than by any given realization of the phase arrangements that has occurred at some point, even if that is the most dominant one. This is especially pronounced when noise is added to the system, making the spectral capacity more informative for the relative activity of the nodes for almost all the frequencies.

### Robustness of the Spectral Strength

**Figure 6.**
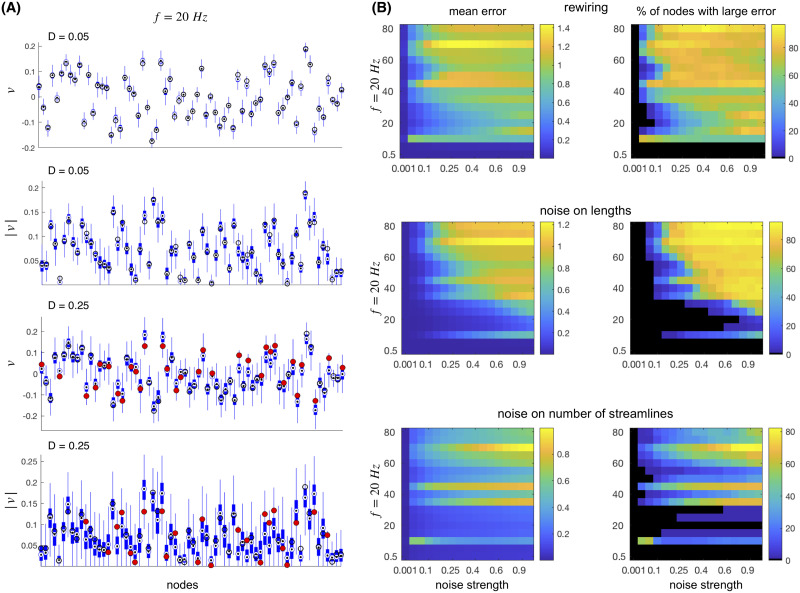
Robustness of the spectral strength. (A) Boxplots showing the statistics of the leading eigenvector and its absolute value for 1,000 realizations of two different levels of noise added to the lengths at *f* = 20 Hz on the connectome of the first subject. Black circles are the values for the original connectome; they are filled red for the cases when the error terms (Equation 7) exceed the error threshold. (B) Mean of the errors (Equation 7) over all the regions shown for different noise and frequencies (left column), as well as the ratio of the nodes whose error exceeds the threshold (right column).

The consistency of the spectral strength of the normalized connectome is tested against different types of uncertainties, which are modeled as rewiring and noise in the connectome, all scaled to have a comparable impact. Boxplots that show the statistics of the leading eigenvector *v* and its absolute value are shown in Figure 6A for one frequency and for two different levels of noise. At lower noise, the averages of the regional activity shown by the eigenvectors of the null model are still following the original values. The variance of the null models’ activity in this case would sufficiently describe the impact of the noise, and the error that it causes. As the noise increases, the variance of the null model also increases, but in addition for some regions the mean starts significantly differing from the original values. This suggests that, to better account for the impact of the noise and the network’s resilience, we need to take into account both the standard deviation *σ*_*i*_ of all realizations of $v_{i}^{\text{null}}$ and the error of their mean ${\bar{v}}_{i}^{\text{null}}$, defined as

$$\tilde{{err}_{i}}=\left|v_{i}-{\bar{v}}_{i}^{\text{null}}\right|.$$ (6)

Moreover, the impact of the above error seems to be inversely proportional to the spread of the results for the null model; that is, the smaller the spread, the larger the chance for the null model to give an error around its mean. Hence, we arrive at the following definition of the error for each node:

$${err}_{i}=\frac{\tilde{{err}_{i}}}{\sigma_{i}}+\frac{\sigma_{i}}{2}.$$ (7)

The mean of those errors over the whole network is shown in the first column of Figure 6B. Finally, not all nodes are equally affected by the noise; to describe this, in the second column of Figure 6B we depict the proportion of the nodes that have an error larger than the threshold set at (*v*_*i*_ + $\bar{v}$)/2. In this way the deviation at each node is evaluated against both the local (i.e., its own) and the global activity.

Results in Figure 6B show that robustness against the noise and rewiring decreases with the frequency, and this is especially evident for the uncertainties of the delays. For frequencies below ∼35 Hz, fewer than half of the nodes have deviations that are considered large for all three types of uncertainties, with rewiring being more sensitive than the noise of the same impact.

## DISCUSSION

Wave metrics offer a new perspective for characterizing dynamic networks. They integrate the wave component of information processing, which was missing in network metrics that were based solely on the connection topology (Bassett & Sporns, 2017). The incorporation of signal transmission delays in the connectome’s metrics completes the characterization of the spatiotemporal skeleton, within which oscillatory brain activity can be amplified by the properties of the medium supporting it, i.e., it provides a *corpus resonantiae*. We propose that the activation of certain parts of the brain, that are related to different tasks, can be explained as being anatomically prewired. We have demonstrated that the connectome has such properties and allows for selective, frequency-dependent information processing that could support the differentiation of brain activity for various processes and frequency bands (Liu et al., 2015; Mejias, Murray, Kennedy, & Wang, 2016). Notably, the wave perspective leads naturally to the formation of overlapping, albeit functionally independent subnetworks, that cannot be derived within the particle picture. Besides revealing the frequency-specific hubness, as another consequence the wave coupling also supports the general reduction of activity with increasing frequencies, which is among the more consistent and well-established features of brain dynamics (Buzsáki & Draguhn, 2004).

The proposed of the connectome is derived from the expressions for the phase lags between synchronized phase oscillators. These are the only dynamical features by which functional heterogeneity can arise in this setup (Moon, Lee, Blain-Moraes, & Mashour, 2015; Petkoski et al., 2018), hidden by structural symmetry breaking of the network interactions (weights and time delays), or from the different natural frequencies. Both of these aspects are present in Equation 3, but in this study, we focus on the former. In that respect we show that in the presence of time delays the weights are effectively scaled (or normalized) by cosΩ*τ*_*ij*_, even though the rest of the network is also needed to fully describe the phase lags and hence the full dynamics. For the case of two oscillators, however, the normalized weights are the sole determinant for the synchronized interactions.

Importantly, however, the network models that we introduced are not intended to serve as toy brain models, but to illustrate the meaning of the metrics derived here. We use established models to expose and justify open and hidden assumptions underlying the traditional and extended metrics (the latter being normalized for time delays in the wave perspective). Every metric relies on an underlying theory or model, even if it is not made explicit. For instance, functional connectivity relies via covariance on second-order metrics (Friston, 2011) adapted to linear models (fitting an ellipsoid to the corresponding data cloud), but this does not preclude its application to nonlinear network models; in fact, in practice, this is often the rule. In the same spirit, we motivate the particle and wave metrics by using rate and Kuramoto oscillator equations, without the claim that these actually represent brain network models.

It is worth noting that studying the synchronizability of the brain networks with time delays could complement our framework for the study of spectrally dependent emergent functional heterogeneity, beyond the forward modeling. A possible approach here would be to use recent extensions of the Ott and Antonsen ansatz (Ott & Antonsen, 2008), that allow the study of noisy networks, either through circular cumulants (Tyulkina, Goldobin, Klimenko, & Pikovsky, 2018) or by using Daido order parameters (Hannay, Forger, & Booth, 2018). These could be applied on the spatially decomposed time delays as a first approximation of the spatiotemporal structure of the connectome (Petkoski et al., 2016). Another direction would be to study the stability of the synchronized states over the network of wave couplings that are both positive and negative (Iatsenko, Petkoski, McClintock, & Stefanovska, 2013).

During tasks, the spectrally dependent activation patterns are also constrained by stimuli. For example, in many visual tasks, they cause gamma synchronization in frontal regions (Roux, Wibral, Mohr, Singer, & Uhlhaas, 2012). This is missing from our predictions at rest and in absence of stimuli. The offered framework also does not account for communication across different frequencies, even though it could be further generalized to other types of *n*:*m* synchronization (Pikovsky et al., 2001). Having data-informed, intrinsic dynamics is also a feature that could increase the predictive value of any generative model for brain dynamics, but this would need to be biased by the specific brain activity (Courtiol, Guye, Bartolomei, Petkoski, & Jirsa, 2020; Schirner, McIntosh, Jirsa, Deco, & Ritter, 2018; Wang et al., 2019). This is, however, still to be generalized in a graph theoretical framework. Similarly, our framework considers the activity at each frequency as fixed—even though they are often time-dependent (Clemson, Lancaster, & Stefanovska, 2016; Petkoski & Stefanovska, 2012; Stankovski, Duggento, McClintock, & Stefanovska, 2012; Stankovski et al., 2016) and intermittent (Sheppard, Hale, Petkoski, McClintock, & Stefanovska, 2013)—and to be a separate process. The latter might lead to omitting certain emergent properties in cases where the same process has clear multimodal frequency content, as is often seen in frequency- and scale-dependent metrics from information theory (Courtiol et al., 2016; Kaminski & Blinowska, 1991; Marinazzo, Pellicoro, & Stramaglia, 2008) and time-frequency representation (Iatsenko, McClintock, & Stefanovska, 2015).

Beyond synchronization, frequency-dependent approaches to information processing offer data-driven insight into causality of network interactions (Reid et al., 2019). Applying transfer entropy (Schreiber, 2000) reveals that high-degree neurons feed cortical computations by receiving more information from high out-degree neurons (Timme et al., 2016), indicating an asymmetry of information transfer between hubs and low-degree nodes (Novelli et al., 2020). Spectral and multiscale Granger causality (Faes, Nollo, Stramaglia, & Marinazzo, 2017; Kaminski & Blinowska, 1991) are similarly used to describe the information flow in networks, and they demonstrate the nonlinear effects of the structural weights, which can be seen through signatures of the law of diminishing marginal returns (Marinazzo et al., 2014; Marinazzo, Wu, Pellicoro, Angelini, & Stramaglia, 2012). It would be interesting to compare the flow that these metrics reveal for the case of synchronization, with the spectrally dependent network core as defined by the normalized connectome.

The predictive value of the normalized connectome for the functional in-strength is further validated through comparisons with numerical simulations of the Stuart-Landau model. This model is often used to describe mesoscopic neuronal activity (Courtiol et al., 2020; Deco et al., 2017; Deco & Kringelbach, 2020; Petkoski & Jirsa, 2019) because it encompasses the working points of population rate models (Deco, Jirsa, McIntosh, Sporns, & Kötter, 2009). Correlating the spatial activation patterns with the predictions from the spectral in-strength and capacity shows that the normalized metrics capture the dynamics of the model better than using only the weights. This is besides the derivations being made on the Kuramoto model that does not contain amplitude dynamics, and that assumes difference instead of additive coupling. Better performance of the wave coupling is consistent across the frequencies, and holds for different working points, such as sub- and supercritical, with or without added noise.

The normalization of the connectome unifies its structural weights and time delays, and consequently it still inherits the known problems of these two modalities. What the number of streamlines means for the actual effective coupling between brain regions regardless of the task still lacks a clear response (Friston, 2011; Gilson et al., 2019). In addition, diffusion tractography is known to undersample the interhemispheric links (Schirner, Rothmeier, Jirsa, McIntosh, & Ritter, 2015) and the topology of the derived connectomes is still dependent on the parcellation (Domhof, Jung, Eickhoff, & Popovych, 2021). The lack of personalized estimations for the time delays of the connectome is still an unavoidable problem. A possible way forward could be using averaged delays derived from stimulation (Trebaul et al., 2018), combined with the personalized lengths of the tracts, even if these datasets are still incomplete. The lack of directionality in the human connectomes that rely on diffusion tractography is also a confounding factor of those connectomes (Melozzi et al., 2019). This has led to introduction of concepts such as effective connectivity (Friston, 2011; Gilson et al., 2019), even though for our framework the derivations are simplified by the symmetry in the coupling strengths.

Finally, we have introduced only two metrics for node activity based on the normalized weights, whereas other graph theoretical metrics for wave interactions could be more informative. However, even for spectral capacity and strength, it is still not clear which one would have higher predictive value in describing the activation patterns of the brain at rest. A systematic and rigorous comparison against empirical data could provide answers about which brain regions operate closer to the limits of their capacity. Similarly, the phase arrangements of the brain do not necessarily need to be such that they maximize the overall information transfer across the whole brain, but maybe instead they give priority to certain areas at the expense of others.

Transient synchronization between brain areas coupled through delayed interactions has been proposed as a mechanism that could support flexible information routing (Palmigiano, Geisel, Wolf, & Battaglia, 2017), which plays a fundamental role in cognition. Similarly, the implementation of selective communication, dependent on coherence, has been generalized to communication through coherence (Fries, 2015) as one of the possible mechanisms of communication between neuronal networks (Hahn, Ponce-Alvarez, Deco, Aertsen, & Kumar, 2019). This offers no explanations of the origin of the selection of frequency bands and the spatial organization of the involved neuronal populations. The normalization of the connectome completes this view mechanistically, hypothesizing that the spatiotemporal brain structure itself could be at least partly responsible for the observed patterns. Through the notion of wave-type communication, the offered normalization lends legitimacy to concepts of synchronization and coherence in network science and opens up a many new applications way beyond brain sciences.

## MODEL AND METHODS

### Particle Versus Wave Representation for Network Dynamics

The particle-wave dichotomy appears in network dynamics because of finite transmission velocities between spatially distributed nodes. Within the particle view, the general network dynamics is governed by the following:

$${\dot{r}}_{i}=g_{1}\left(r_{i}\right)+\sum_{j}w_{ij}g_{2}\left(r_{j}\left(t-\tau_{ij}\right)\right).$$ (8)

Here *r*_*i*_ is any scalar variable such as the firing rate, *w*_*ij*_ are the weights and *τ*_*ij*_ are the time delays of the links, and *g*_1_ and *g*_2_ are linear functions for the intrinsic dynamics and coupling, respectively. This type of particle-like interaction is frequency-independent, and here the information and the activity always flow along the links with the strongest coupling weight.

On the other hand, networks of oscillators are often used to conceptualize and to study dynamical systems for which the local activity is multidimensional and nonlinear (Pikovsky et al., 2001). They have been conceptualized to be responsible for the communication in the brain through coherence (Fries, 2015) and synchronization (Kirst, Timme, & Battaglia, 2016; Palmigiano et al., 2017; Palva & Palva, 2012). In this case the dynamics of the oscillatory network nodes are governed by Equation 1. When delays are negligible compared with the timescale of the system, the interactions are still governed only by the weights, as in the particle case. However, when the time delays are comparable to the timescale of the intrinsic oscillations, they need to be included in the analysis of network dynamics. As a consequence, this system can only be solved numerically, as there is no alternative graph theoretical approach that integrates the topology and the impact of the time delays on the oscillations at a given frequency. Hence the systematic effect of the time delays on the emergent dynamics is concealed, and even the full representation of interactions in Equation 1, with separated *w*_*ij*_ and *τ*_*ij*_, cannot adequately identify the skeleton of the wave synergy.

For weak couplings, dynamics of the system in Equation 1 are captured by phase models, which are often used to study the interactions in the brain at different levels (Sheppard et al., 2013; Stankovski et al., 2016). The simplest and hence most elaborate is the Kuramoto model (Kuramoto, 1984). It is widely utilized for describing emergent phenomena in complex systems (Strogatz, 2003), such as the brain (Allegra Mascaro et al., 2020; Breakspear, Heitmann, & Daffertshofer, 2010; Cabral, Hugues, Sporns, & Deco, 2011), with a structure often represented via complex networks (Rodrigues, Peron, Ji, & Kurths, 2016). For synchronization at frequency Ω, the model (Izhikevich, 1998) reads as follows:

$${\dot{\theta }}_{i}=\omega_{i}-\sum_{j}w_{ij}\sin\left(\theta_{i}-\theta_{j}\left(t-\tau_{ij}\right)\right)=\omega_{i}-\sum_{j}w_{ij}\sin\left(\theta_{i}-\theta_{j}+\Omega \tau_{ij}\right),$$ (9)

where *θ*_*i*_ denotes the phase of the *i*th node oscillating with a natural frequency *ω*_*i*_, the dependence on time is implicit, and because of the synchronization *θ*_*j*_(*t* − *τ*_*ij*_) = *θ*_*j*_(*t*) + Ω*τ*_*ij*_. From here, the phase difference between each pair of Kuramoto oscillators is given as

$$\begin{array}{l} {\dot{\theta }}_{ij}={\dot{\theta }}_{i}-{\dot{\theta }}_{j}=\Omega -\Omega =\omega_{i}-\omega_{j}-\sum_{k}w_{ik}\sin\left(\theta_{i}-\theta_{k}+\Omega \tau_{ik}\right)+\sum_{k}w_{jk}\sin\left(\theta_{j}-\theta_{k}+\Omega \tau_{jk}\right)\\ \;=2\Delta \omega_{ij}-\sum_{k}\left(w_{ik}\sin\left(\theta_{i}-\theta_{k}+\Omega \tau_{ik}\right)+w_{jk}\sin\left(\theta_{j}-\theta_{k}+\Omega \tau_{jk}\right)\right), \end{array}$$ (10)

where Δ*ω*_*ij*_ = (*ω*_*i*_ − *ω*_*j*_)/2. Next, we introduce Δ*θ*_*ij*_ = *θ*_*i*_ − *θ*_*j*_ and we separate the sum, such that

$$\begin{array}{l} 0=2\Delta \omega_{ij}-w_{ij}\sin\left(\Delta \theta_{ij}+\Omega \tau_{ij}\right)+w_{ji}\sin\left(\Delta \theta_{ji}+\Omega \tau_{ji}\right)\\ \;-\sum_{k\neq i,j}\left(w_{ik}\sin\left(\Delta \theta_{ik}+\Omega \tau_{ik}\right)-w_{jk}\sin\left({\Delta \theta }_{jk}+\Omega \tau_{jk}\right)\right)\\ \;=2\Delta \omega_{ij}-w_{ij}\sin\left(\Delta \theta_{ij}+\Omega \tau_{ij}\right)+w_{ji}\sin\left(\Delta \theta_{ji}+\Omega \tau_{ji}\right)-2I_{ij}, \end{array}$$ (11)

where

$$I_{ij}=\frac{1}{2}\sum_{k\neq i,j}\left(w_{ik}\sin\left(\Delta \theta_{ik}+\Omega \tau_{ik}\right)-w_{jk}\sin\left({\Delta \theta }_{jk}+\Omega \tau_{jk}\right)\right).$$ (12)

Note that *I*_*ij*_ contains all the other links towards the nodes *i* and *j* apart from their direct link.

For symmetric coupling, *w*_*ij*_ = *w*_*ji*_ and *τ*_*ij*_ = *τ*_*ji*_, while Δ*θ*_*ij*_ = Δ*θ*_*ij*_ by definition, hence Equation 11 becomes

$$\begin{array}{l} 0=2\Delta \omega_{ij}-w_{ij}\left[\sin\left(\Delta \theta_{ij}+\Omega \tau_{ij}\right)-\sin\left(-\Delta \theta_{ij}+\Omega \tau_{ij}\right)\right]-2I_{ij}\\ \;=2{\Delta \omega }_{ij}-2w_{ij}\sin\Delta \theta_{ij}\cos\Omega \tau_{ij}-2I_{ij}. \end{array}$$ (13)

Finally, for the phase difference between the oscillators we get

$${\Delta \theta }_{ij}=\sin^{-1}\frac{\Delta \omega_{ij}-I_{ij}}{w_{ij}\cos\Omega \tau_{ij}}=\sin^{-1}\frac{\Delta \omega_{ij}-I_{ij}}{w_{ij}^{\left(f\right)}},$$

where $w_{ij}^{\left(f\right)}$ = *w*_*ij*_ cosΩ*τ*_*ij*_ is the normalized weight containing the impact of the direct link between the pair of oscillators. For particle-like communication in the limit Ω*τ*_*ij*_ → 0, the cosine term and *I*_*ij*_ vanish, making the coupling weights the only factor shaping the network dynamics, consistent with the case of zero delays. Numerical simulations validate the analytical results for Δ*θ*_*ij*_ by showing that the difference between the analytical and numerical values is small and decreases for smaller integration steps, and increases with added noise (Figure S1 in the Supporting Information).

In the case of asymmetric weights, Equation 13 would also include a term that accounts for the asymmetry in the interactions *w*_*ij*_ − *w*_*ji*_, and the exact derivation of the expression in Equation 3 will not be possible anymore. However, if the asymmetry is relatively small, as is the case for the mice tracer connectome (Oh et al., 2014) then the same expression would approximately hold up to a leading term. As for the human connectomes, they almost exclusively rely on diffusion tractography imaging, which produces links with symmetric weights.

### Limits of the Approximation

If we assume a normalized connectome, the governing equation of the phase dynamics becomes

$${\dot{\theta }}_{i}=\omega_{i}-\sum_{j}w_{ij}\cos\Omega \tau_{ij}\sin\left(\theta_{i}-\theta_{j}\right).$$ (14)

Following the same steps as above, the phase difference between a pair of oscillators reads

$$\begin{array}{l} {\dot{\theta }}_{i}-{\dot{\theta }}_{j}=\Omega -\Omega =2\Delta \omega_{ij}-\sum_{k}\left(w_{ik}\cos\Omega \tau_{ik}\sin\Delta \theta_{ik}-w_{jk}\cos\Omega \tau_{jk}\sin\Delta \theta_{jk}\right)\\ \;=2\Delta \omega_{ij}-2w_{ij}\cos\Omega \tau_{ij}\sin\Delta \theta_{ij}-\sum_{k\neq i,j}\left(w_{ik}\cos\Omega \tau_{ik}\sin\Delta \theta_{ik}-w_{jk}\cos\Omega \tau_{jk}\sin{\Delta \theta }_{jk}\right)\\ \;=2\Delta \omega_{ij}-2w_{ij}\cos\Omega \tau_{ij}\sin\Delta \theta_{ij}-2J_{ij}. \end{array}$$ (15)

As for the difference between the full model and the approximation using the normalized weights, we get

$$\begin{array}{l} I_{ij}-J_{ij}=\frac{1}{2}\sum_{k\neq i,j}\left(w_{ik}\sin\left(\Delta \theta_{ik}+\Omega \tau_{ik}\right)-w_{jk}\sin\left({\Delta \theta }_{jk}+\Omega \tau_{jk}\right)-w_{ik}\cos\Omega \tau_{ik}\sin\Delta \theta_{ik}+w_{jk}\cos\Omega \tau_{jk}\sin{\Delta \theta }_{jk}\right)\\ \;=\frac{1}{2}\sum_{k\neq i,j}\left(w_{ik}\sin\Omega \tau_{ik}\cos\Delta \theta_{ik}-w_{jk}\sin\Omega \tau_{jk}\cos\Delta \theta_{jk}\right). \end{array}$$ (16)

For many symmetric networks, such as the delay-imposed clusters with identical weights (Petkoski et al., 2016), this difference vanishes.

### Forward Modeling and Linear Stability Analysis

The normalization of the connectome also allows straightforward application of LSA for the dynamics close to a critical state for a given forward model of the oscillatory activity. To build our brain network model (Courtiol et al., 2020; Olmi et al., 2019; Petkoski & Jirsa, 2019; Petkoski et al., 2018) over the same 100 human connectomes, we choose Stuart-Landau oscillators, as a canonical model for oscillations near Andronov-Hopf bifurcation (Kuramoto, 1984), which is also the working point of many neuronal mass models (Deco & Jirsa, 2012; Kringelbach & Deco, 2020). The local dynamics from Equation 1 are then given as

$$F\left(X_{i}\right)=X_{i}\left(r+j\omega -{\left|X_{i}\right|}^{2}\right),$$ (17)

where *X*_*i*_ is a complex number, and the coupling is linear additive with

$$w_{ij}h\left(X_{j}\left(t-\tau_{ij}\right)\right)=w_{ij}^{\left(f\right)}X_{j}.$$ (18)

All the parameters, including the frequency *ω* and the distance from the local bifurcation *r*, are identical for every oscillator. The stability of this system can be analyzed by following the evolution of infinitesimal perturbations in the tangent space, whose dynamics are ruled by the linearization of Equation 1 with Equations 17 and 18. If the eigenvalues of the Jacobian matrix all have real parts less than 0, then the steady state is stable. For each frequency for which the system is analyzed, we set the parameter *r* to keep the system at the edge of criticality. This is done in an iterative procedure, where *r* is decreased until the system is just above the criticality. In this case there are two identical positive eigenvalues, which contain the same eigenvectors of opposite signs, because of the way that the two dimensions of the local model are coupled.

In addition to the LSA, we carried a numerical simulation over the full connectome of the same model, with the coupling term reading

$$w_{ij}h\left(X_{j}\left(t-\tau_{ij}\right)\right)=Gw_{ij}X_{j}\left(t-\tau_{ij}\right),$$ (19)

where *G* is a global coupling parameter. Numerical integration is performed using stochastic Heun integration scheme implemented in MATLAB, with an integration time step that is inversely proportional to the natural frequencies and the global coupling, but it is never larger than 0.005 s. In some of the analyzed scenarios the dynamics also contain white additive Gaussian, with noise intensity *D*. The critical value of *r* is different for each connectome and each frequency, and sub- and supercriticality are set such that *r* is at 0.2 below or above criticality, respectively.

### Setting the Weights for the Coupling

Human connectomes were derived from the first release of diffusion tractography imaging of 100 healthy subjects, which is publicly available as part of the Human Connectome Project (Van Essen et al., 2013). The subjects were scanned on a customized 3T scanner at Washington University, and the structural connectivity was constructed using a publicly available pipeline (Tournier et al., 2019) that applies a spherical deconvolution method to a probabilistic streamline tracking algorithm. The obtained connectome consist of few million tracts spatially averaged to connect 68 cortical regions defined according to the Desikan-Killiany atlas (Desikan et al., 2006). For each link, weights are numbers of individual tracts between the regions of that link, and lengths are their averages.

The mouse connectome in Figure 2A was obtained from the Allen Institute Mouse Brain Connectivity data (Oh et al., 2014), which is integrated in The Virtual Mouse Brain (Melozzi, Woodman, Jirsa, & Bernard, 2017), and which was already used to model mice brain dynamics (Allegra Mascaro et al., 2020; Melozzi et al., 2019).

The spectral graph theoretical results (Figures 2–4) and the LSA analysis (Figures 5A and 6) use the natural logarithms of number of streamlines as weights of the couplings. This is done for better illustration of the findings in order to decrease the heterogeneity between the different nodes. For the full forward model (Figure 5B), we use the number of streamlines, as it has been more common in the numerical simulations (Courtiol et al., 2020; Olmi et al., 2019; Petkoski & Jirsa, 2019; Petkoski et al., 2018).

### Phase Arrangement of the Nodes That Maximizes the Spectral Strength

When the in-/anti-phase arrangement of the nodes is such that *S*^(*f*)^ is maximized, this is referred to as $S_{\text{conn}}^{\left(f\right)}$, since the arrangement takes into account only the connectome, but not the model. Unless specified otherwise in the text, spectral strength *S*^(*f*)^ refers to $S_{\text{conn}}^{\left(f\right)}$. The clusters of in-/anti-phase nodes can be of any size, but the assigning of the clusters is not important (e.g., *i* ∈ *Q*_*i*_ ≡ {1, 2, …, *N*}\*i* ∈ *Q*_*i*_, and similarly swapping all the red and blue colors in Figure 1 does not change the results). As a consequence, the total number of combinations is ∑k=1N/2Nk ≈ $\frac{N!}{2}$ and a brute force procedure cannot be used to identify the clustering that yields the maximum $S_{\text{conn}}^{\left(f\right)}$. Hence, we developed a heuristic procedure that seems to be able to identify the clusters of maximum spectral strength. In short, it starts with random initial arrangements and then uses three types of cost functions to identify the node that needs to change the cluster. These are the following:

-  The node with the smallest *S*^(*f*)^ changes the cluster.
-  The node with a smaller *S*^(*f*)^ from the two nodes of the link that has the smallest (most negative) internal (from the links within each cluster) $w_{ij}^{\left(f\right)}$.
-  The node with a smaller *S*^(*f*)^ from the two nodes of the link that has the smallest (most negative) external (from the links between the clusters) $w_{ij}^{\left(f\right)}$.

For better sampling of different arrangements, a randomness is also systematically added in some of the loops, so that the cost function chooses a suboptimal node or link among some proportion of them that were identified to have the largest cost.

The procedure is repeated until it recovers a cluster that has been already identified. At the end of the runs, the largest spectral capacity that was obtained is set to be $S_{\text{conn}}^{\left(f\right)}$. In particular, we start from 10 random initializations for the phases, and for each of them the three procedures are repeated one after another until an already visited configuration is achieved, after which the loop is repeated 10 more times with added randomness. Every loop is stopped after arriving at an already visited configuration, and all the visited configurations are then classified according to the highest spectral strength. We verified that the convergence to the selected configuration occurs even after 10–100 times fewer samples than we generate with our procedure.

### Projecting the Spectral Strengths and Capacities Into RSNs and Brain Lobes

To better illustrate the patterns of spectral activity, we project the spectral strength and capacity in each brain lobe and RSN (Figures 2–4). For each subject, after calculating them for a given frequency (from 0 to 80 Hz with a step of 1 Hz), we calculate the average (per number of regions in the given subnetwork) activity of a given subnetwork (RSN or lobe). In addition, for the results in Figures 3 and 4, the values of each subnetwork are normalized at each frequency, to focus on the relative activation patterns. The activity of different frequency bands contains the averaged activity of the frequencies within that band (see Table S13 in the Supporting Information). The mean and the standard deviation over the subjects are calculated for each frequency for the activity of the brain lobes.

Note that averaging over subjects in Figure 4B is done over the spectral strengths, which explicitly take into account the in-/anti-phase arrangements by assigning them opposite signs. In particular, the regions that are in-phase with regions that have strongest spectral strength over the subjects are set to be always of a positive sign (blue color), as compared with the others that are negative (red). Since during the averaging some nodes can be of opposite phase arrangements over the subjects, the absolute value of the observed activity here can still be different from the one shown in Figure 4A.

### Robustness of the Spectral Strength

To test the robustness of the results, we modified connectomes by either introducing noise or rewiring. We use three types of modified connectomes:

-  Noise is added to the lengths of the links, with the strength proportional to the mean length of the links.
-  Noise is added to the number of streamlines that are used for calculating the weights. The level of the noise is proportional to the mean number of streamlines of the links.
-  A proportion of the links equivalent to the level of noise is rewired while preserving the distribution of in-strengths (Rubinov & Sporns, 2011).

For each of these and for each frequency, we perform 1,000 realizations of the null networks, and then we compute the LSA for each of them to obtain the patterns of activity.

## DATA AVAILABILITY

All the data, codes, and relevant information to reproduce the results in the paper are available in a public repository; see https://github.com/esaps/NormConn (Petkoski, 2022).

## SUPPORTING INFORMATION

Supporting information for this article is available at https://doi.org/10.1162/netn_a_00231.

## ACKNOWLEDGMENT

We are grateful to Kashyap Gudibanda for his useful comments on the manuscript.

## AUTHOR CONTRIBUTIONS

Spase Petkoski: Conceptualization; Formal analysis; Investigation; Methodology; Software; Validation; Visualization; Writing – original draft. Viktor K. Jirsa: Conceptualization; Methodology; Project administration; Resources; Supervision; Validation; Writing – original draft.

## FUNDING INFORMATION

Viktor K. Jirsa, Horizon 2020 Framework Programme (https://dx.doi.org/10.13039/100010661), Award ID: 826421. Viktor K. Jirsa, Horizon 2020 (https://dx.doi.org/10.13039/501100007601), Award ID: 945539. Viktor K. Jirsa, Horizon 2020, Award ID: 785907.

## TECHNICAL TERMS

Synchronization: Coordinated activity due to interactions, resulting in network nodes oscillating with identical frequency.
Oscillator: A system capable of generating stable rhythmic activity. From a dynamical viewpoint, it has to contain at least two dimensions (variables) and some nonlinear interactions.
Space-time (or spatiotemporal) structure of the brain: Unified consideration of the topology (weights) and space (time-delays) of a given network.
Emergent dynamics: Behaviors that emerge only when the interacting subsystems are considered as a whole (e.g., synchronization can emerge when interacting oscillators are considered to be an ensemble).
Mechanistic explanation: Description that offers testable causal links for generating certain phenomena.
Synchronizability: Easiness/propensity for a population to get synchronized.
Static network: A network representation that is invariant on identical shift of the frequencies, thus being ignorant of the absolute frequencies.
Normalization: Adjusting a quantity (structural weight in our case) to compensate for an additional effect on it by another quantity (time delays during synchronization in our case).
Phase oscillator: An oscillator whose amplitude of oscillations is constant, leading to one-dimensional representation in polar coordinates.

## Supporting Information

https://github.com/esaps/NormConn
