# Supplementary material for: Normalizing the brain connectome for communication through synchronization
Source: Netw Neurosci. 2022 Jul 1;6(3):722–44. doi: 10.1162/netn_a_00231 (PMC9810372; doi:10.1162/netn_a_00231)
Supplement: Supplementary file 1 [file netn-06-722-s001.pdf]

Petkoski, S. & Jirsa, V. K. (2022). Supporting Information for "Normalizing the brain connectome for communication through synchronization." *Network Neuroscience 6*(3), 722–744.
https://doi.org/10.1162/netn_a_00231

Supplemental Material for

Normalizing the brain connectome for communication through synchronization

S Petkoski, VK Jirsa.

Correspondence to: spase.petkoski@univ-amu.fr & viktor.jirsa@univ-amu.fr

This document includes:

Figure S1

Tables S1 to S15

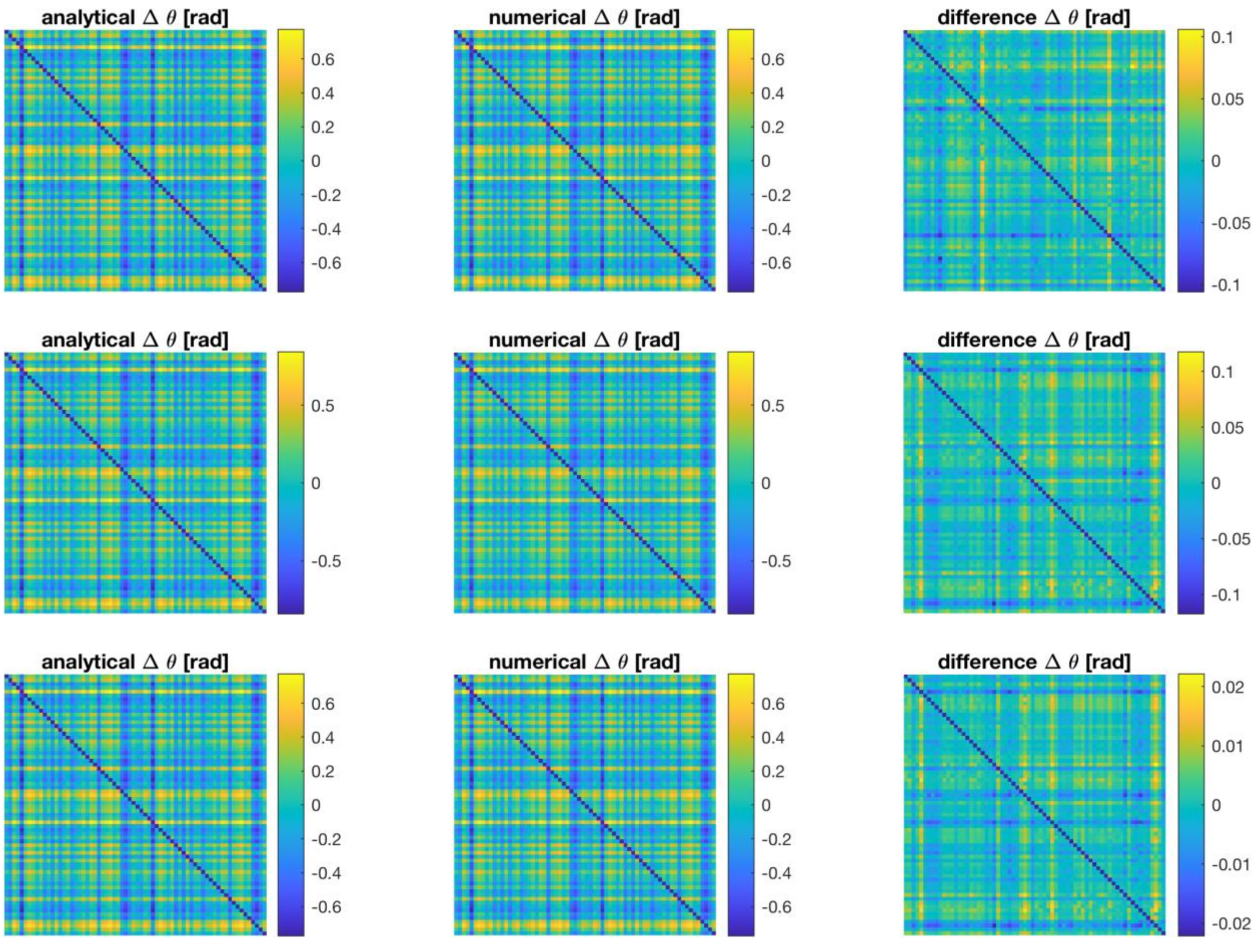

Fig. S1. Analytical (Eq. 3) and numerical results for the phase difference between each pair of brain regions of a single subject, for simulation of the Kuramoto model with (upper row) time step dt=0.0005 and noise intensity D=0, (middle row) dt=0.0001 and D=0.2, and (bottom row) dt=0.0001 and D=. A constant weight of 0.01 is added to all the weights of the connectome, so that all the nodes are synchronized, while preserving the heterogeneity. Parameters: f=10Hz, v=3.3m/s, G=3.

**delta**	**Visual**	**SensMot**	**Auditory**	**ExecCont**	**FronPar**
**DMN**	9.4e-9	2.4e-41	2.85e-16	0.14	0.21
**Visual**	-	1.7e-55	4.5e-32	3.1e-05	2.37e-07
**SensMot**	-	-	1.1e-13	7.7e-43	5.5e-49
**Auditory**	-	-	-	6.6e-19	6.7e-22
**ExecCont**	-	-	-	-	0.67

Table S1. p values of the t-test for the null hypothesis that the normalized spectral content in the delta frequency band in different RSNs comes from independent random samples from normal distributions with equal means and equal but unknown variances.

theta	Visual	SensMot	Auditory	ExecCont	FronPar
**DMN**	5.5e-05	8.1e-34	2.3e-19	9.7e-13	3.5e-34
**Visual**	-	6.4e-41	1.2e-27	6.3e-20	1.3e-14
**SensMot**	-	-	9.1e-05	2.8e-04	1.7e-73
**Auditory**	-	-	-	0.70	4.9e-59
**ExecCont**	-	-	-	-	4.6e-43

Table S2. p values of the t-test for the null hypothesis that the normalized spectral content in the theta frequency band in different RSNs comes from independent random samples from normal distributions with equal means and equal but unknown variances.

alpha	Visual	SensMot	Auditory	ExecCont	FronPar
**DMN**	7.6e-101	2.1e-75	9.4e-30	0.53	2.4e-56
**Visual**	-	4.7e-144	7.0e-82	2.8e-84	5.3e-16
**SensMot**	-	-	6.1e-107	2.4e-60	2.3e-107
**Auditory**	-	-	-	1.0e-22	2.2e-30
**ExecCont**	-	-	-	-	1.98e-49

Table S3. p values of the t-test for the null hypothesis that the normalized spectral content in the alpha frequency band in different RSNs comes from independent random samples from normal distributions with equal means and equal but unknown variances.

beta	Visual	SensMot	Auditory	ExecCont	FronPar
**DMN**	5.4e-05	2.1e-35	1.9e-56	1.6e-06	4.0e-4
**Visual**	-	3.3e-26	4.6e-45	1.26e-15	7.3e-15
**SensMot**	-	-	0.40	2.2e-44	7.5e-47
**Auditory**	-	-	-	2.1e-63	2.47e-76
**ExecCont**	-	-	-	-	2.0e-2

Table S4. p values of the t-test for the null hypothesis that the normalized spectral content in the beta frequency band in different RSNs comes from independent random samples from normal distributions with equal means and equal but unknown variances.

low gamma	Visual	SensMot	Auditory	ExecCont	FronPar
**DMN**	3.1e-3	2.5e-2	2.5e-22	0.19	9.0e-18
**Visual**	-	0.86	4.03e-31	4.4e-4	1.0e-10
**SensMot**	-	-	2.5e-24	3.0e-3	4.4e-08
**Auditory**	-	-	-	7.4e-13	2.8e-47
**ExecCont**	-	-	-	-	5.2e-15

Table S5. p values of the t-test for the null hypothesis that the normalized spectral content in the low gamma frequency band in different RSNs comes from independent random samples from normal distributions with equal means and equal but unknown variances.

gamma	Visual	SensMot	Auditory	ExecCont	FronPar
**DMN**	2.6e-08	7.3e-21	3.7e-14	9.4e-09	1.9e-2
**Visual**	-	2.1e-31	3.9e-2	0.67	6.0e-4
**SensMot**	-	-	4.3e-37	3.2e-31	7.4e-25
**Auditory**	-	-	-	0.12	3.6e-08
**ExecCont**	-	-	-	-	2.0e-4

Table S6. p values of the t-test for the null hypothesis that the normalized spectral content in the gamma frequency band in different RSNs comes from independent random samples from normal distributions with equal means and equal but unknown variances.

DMN	theta	alpha	beta	low gamma	gamma
**delta**	0.11	1.1e-30	2.3e-15	2.2e-2	5.6e-4
**theta**	-	1.8e-34	1.9e-11	0.38	0.057
**alpha**	-	-	3.0e-49	1.0e-32	6.1e-39
**beta**	-	-	-	8.5e-08	2.4e-07
**low gamma**	-	-	-	-	0.43

Table S7. p values of the t-test for the null hypothesis that the normalized spectral content in the Default Mode Network in different frequencies comes from independent random samples from normal distributions with equal means and equal but unknown variances.

Visual	theta	alpha	beta	low gamma	gamma
**delta**	8.3e-03	3.6e-95	4.3e-18	8.2e-06	4.7e-02
**theta**	-	2.4e-84	1.6e-09	9.4e-02	0.66
**alpha**	-	-	4.3e-72	3.5e-83	3.2e-80
**beta**	-	-	-	2.9e-06	1.6e-09
**low gamma**	-	-	-	-	4.5e-02

Table S8. p values of the t-test for the null hypothesis that the normalized spectral content in the Visual network in different frequencies comes from independent random samples from normal distributions with equal means and equal but unknown variances.

SensMot	theta	alpha	beta	low gamma	gamma
**delta**	0.76	6.1e-127	3.2e-53	4.6e-29	0.88
**theta**	-	1.2e-124	1.7e-51	1.7e-27	0.92
**alpha**	-	-	1.5e-68	6.5e-91	1.9e-112
**beta**	-	-	-	5.8e-13	9.2e-45
**low gamma**	-	-	-	-	8.0e-23

Table S9. p values of the t-test for the null hypothesis that the normalized spectral content in the Sensory Motor network in different frequencies comes from independent random samples from normal distributions with equal means and equal but unknown variances.

Auditory	theta	alpha	beta	low gamma	gamma
**delta**	2.5e-03	7.7e-14	3.7e-59	2.6e-10	8.1e-27
**theta**	-	6.47e-21	7.0e-63	1.6e-04	4.8e-33
**alpha**	-	-	9.5e-35	1.5e-28	1.4e-07
**beta**	-	-	-	7.8e-64	3.6e-12
**low gamma**	-	-	-	-	4.6e-39

Table S10. p values of the t-test for the null hypothesis that the normalized spectral content in the Auditory network in different frequencies comes from independent random samples from normal distributions with equal means and equal but unknown variances.

**ExecCont**	**theta**	**alpha**	**beta**	**low gamma**	**gamma**
**delta**	4.8e-18	2.5e-19	3.9e-30	1.2e-4	4.1e-02
**theta**	-	1.5e-37	2.2e-02	5.8e-05	7.6e-20
**alpha**	-	-	4.3e-47	4.3e-23	1.6e-12
**beta**	-	-	-	5.3e-10	3.3e-30
**low gamma**	-	-	-	-	7.6e-07

Table S11. p values of the t-test for the null hypothesis that the normalized spectral content in the Executive Control network in different frequencies comes from independent random samples from normal distributions with equal means and equal but unknown variances.

FronPar	theta	alpha	beta	low gamma	gamma
**delta**	5.1e-30	6.8e-36	4.3e-41	2.9e-13	4.6e-02
**theta**	-	1.1e-55	1.2e-75	1.8e-02	3.1e-27
**alpha**	-	-	2.9e-09	2.3e-46	5.6e-29
**beta**	-	-	-	4.4e-52	5.6e-25
**low gamma**	-	-	-	-	9.2e-15

Table S12. p values of the t-test for the null hypothesis that the normalized spectral content in the Frontal Parietal network in different frequencies comes from independent random samples from normal distributions with equal means and equal but unknown variances.

Frequency bands	delta	theta	alpha	beta	low gamma	gamma
**freq. [Hz]**	1-4	4-8	8-13	13-30	30-50	50-80

Table S13. EEG frequency band

**Lobes**	Regions
**Frontal**	caudalmiddlefrontal, lateralorbitofrontal, medialorbitofrontal, paracentral, parsopercularis, parsorbitalis, parstriangularis, precentral, rostralmiddlefrontal, superiorfrontal, frontalpole
**Cingulate**	caudalanteriorcingulate, isthmuscingulate, posteriorcingulate, rostralanteriorcingulate
**Parietal**	inferiorparietal, postcentral, precuneus, superiorparietal, supramarginal
**Temporal**	bankssts, entorhinal, fusiform, inferiortemporal, middletemporal, parahippocampal, superiortemporal, temporalpole, transversetemporal, insula
**Occipital**	cuneus, lateraloccipital, lingual, pericalcarine

Table S14. Brain regions of different lobes with same ordering as in Figs. Fig. 2 (B) and 3.

Restion State Networks	Regions
**DMN**	inferiorparietal, isthmuscingulate, medialorbitofrontal, posteriorcingulate, precuneus, frontalpole
**Visual**	cuneus, fusiform, lateraloccipital, lingual, pericalcarine, precuneus
**Sensory Motor**	paracentral, postcentral, posteriorcingulate, precentral, paracentral
**Auditory**	bankssts, parstriangularis, superiortemporal, transversetemporal, insula
**Executive Control**	caudalanteriorcingulate rostralanteriorcingulate, rostralmiddlefrontal
**Frontal Parietal**	caudalmiddlefrontal, inferiorparietal, parsopercularis, parsorbitalis, parstriangularis, rostralmiddlefrontal, superiorparietal, supramarginal

Table S15. Brain regions of different Resting State Networks (RSNs).
